# Supplementary material for: Psychological distress, employment, and family functioning during the COVID-19 outbreak among recent immigrant families in Israel: Moderating roles of COVID-19 prevalence
Source: PLoS One. 2022 Nov 17;17(11):e0277757. doi: 10.1371/journal.pone.0277757 (PMC9671308; doi:10.1371/journal.pone.0277757)
Supplement: S1 Table — (DOCX) [file pone.0277757.s001.docx]

**S1 Table.** **Correlations among COVID-19 distress, employment status, daily prevalence, and family functioning indicators.**

|  | 1 | 2 | 3 | 4 | 5 | 6 | 7 | 8 | 9 | 10 | 11 | 12 | 13 | 14 |
| --- | --- | --- | --- | --- | --- | --- | --- | --- | --- | --- | --- | --- | --- | --- |
| **Family functioning** |  |  |  |  |  |  |  |  |  |  |  |  |  |  |
| *Parents* |  |  |  |  |  |  |  |  |  |  |  |  |  |  |
| 1. P-A communication | ̶ |  |  |  |  |  |  |  |  |  |  |  |  |  |
| 1. Parental Involvement | .51^***^ | ̶ |  |  |  |  |  |  |  |  |  |  |  |  |
| 1. Positive parenting | .31^***^ | .51^***^ | ̶ |  |  |  |  |  |  |  |  |  |  |  |
| 1. Family conflict | -.31^***^ | -.28^***^ | -.16^*^ | ̶ |  |  |  |  |  |  |  |  |  |  |
| *Adolescents* |  |  |  |  |  |  |  |  |  |  |  |  |  |  |
| 1. P-A communication | .32^***^ | .29^***^ | .17^*^ | -.07 | ̶ |  |  |  |  |  |  |  |  |  |
| 1. Parental Involvement | .35^***^ | .57^***^ | .37^***^ | -.09 | .66^***^ | ̶ |  |  |  |  |  |  |  |  |
| 1. Positive parenting | .36^***^ | .40^***^ | .37^***^ | -.08 | .57^***^ | .69^***^ | ̶ |  |  |  |  |  |  |  |
| 1. Family conflict | -.30^***^ | -.29^***^ | -.24^**^ | .36^***^ | -.51^***^ | -.48^***^ | -.46^***^ | ̶ |  |  |  |  |  |  |
| **COVID-19 Distress** |  |  |  |  |  |  |  |  |  |  |  |  |  |  |
| 1. Parents | -.05 | .03 | -.06 | .16^*^ | .06 | .04 | -.08 | .11 | ̶ |  |  |  |  |  |
| 1. Adolescents | -.14^†^ | -.18^*^ | -.11 | -.03 | -.07 | -.16^*^ | -.22^**^ | .17^*^ | .17^*^ | ̶ |  |  |  |  |
| **Employment Status** |  |  |  |  |  |  |  |  |  |  |  |  |  |  |
| 1. Stopped working | .03 | -.01 | -.11 | .17^*^ | .04 | .01 | -.08 | .06 | .06 | -.05 | ̶ |  |  |  |
| 1. Working from home | .19^*^ | .07 | .05 | -.09 | -.14 | -.07 | -.12 | .08 | -.01 | -.08 | .01 | ̶ |  |  |
| **COVID Prevalence** |  |  |  |  |  |  |  |  |  |  |  |  |  |  |
| 1. New cases | -.17^*^ | -.08 | -.04 | .07 | -.17^*^ | -.21^**^ | -.14^*^ | .14^*^ | -.10 | .07 | -.06 | -.04 | ̶ |  |
| 1. Death | .06 | .03 | -.06 | -.06 | .13 | .12 | .04 | -.18^*^ | -.08 | .00 | -.09 | .07 | .68^***^ | ̶ |

P-A communication = Parent–Adolescent communication.
†*p*<.10. **p*<.05. ***p*<.01. ****p*<.001.
