# Supplementary material for: Psychological distress, employment, and family functioning during the COVID-19 outbreak among recent immigrant families in Israel: Moderating roles of COVID-19 prevalence
Source: PLoS One. 2022 Nov 17;17(11):e0277757. doi: 10.1371/journal.pone.0277757 (PMC9671308; doi:10.1371/journal.pone.0277757)
Supplement: S2 Table — (DOCX) [file pone.0277757.s002.docx]

**S2 Table.** **Means or Correlations of Family Functioning among Participants’ Demographic Characteristics.**

|  | P-A Communication | Parental Involvement | Positive parenting | Family conflict |
| --- | --- | --- | --- | --- |
| Variables | M (SD) or *r* | M (SD) or *r* | M (SD) or *r* | M (SD) or *r* |
| ***Parents ^a^*** |  |  |  |  |
| Gender |  |  |  |  |
| Male | 3.80 (.62) | ***3.92 (.51)*** | ***3.91 (.70)*** | 2.28 (.51) |
| Female | 3.93 (.58) | ***4.31 (.43)*** | ***4.27 (.48)*** | 2.25 (.49) |
| Age | .68 | -.05 | -.10 | -.05 |
| Years of immigration to Israel | .12 | .02 | .05 | .07 |
| Education level |  |  |  |  |
| High school (including not completed) | 3.95 (.56) | 4.33 (.41) | 4.29 (.47) | 2.24 (.53) |
| College (including graduate college) | 3.87 (.62) | 4.17 (.50) | 4.13 (.58) | 2.26 (.44) |
| Marital Status |  |  |  |  |
| Married | 3.90 (.57) | 4.27 (.46) | 4.22 (.53) | 2.26 (.49) |
| Divorced / Living separately / Never married | 4.00 (.63) | 4.22 (.47) | 4.27 (.48) | 2.20 (.51) |
| Family income (unit: Shekel) |  |  |  |  |
| Less than 10,000 | 3.97 (.62) | 4.25 (.50) | 4.26 (.54) | 2.24 (.51) |
| 10,000 to 15,000 | 3.84 (.58) | 4.28 (.42) | 4.16 (.53) | 2.22 (.47) |
| Over 15,000 | 3.92 (.44) | 4.27 (.34) | 4.28 (.43) | 2.39 (.47) |
| People to room ratio | -.06 | .03 | -.06 | .05 |
| Time social distancing |  |  |  |  |
| No | 3.97 (.55) | 4.28 (.42) | 4.23 (.51) | 2.26 (.47) |
| One month or less | 3.78 (.93) | 4.52 (.54) | 4.40 (.68) | 2.20 (.28) |
| More than one month | 3.79 (.63) | 4.16 (.50) | 4.18 (.55) | 2.25 (.54) |
| ***Adolescents ^b^*** |  |  |  |  |
| Gender |  |  |  |  |
| Male | 3.74 (.60) | 3.84 (.59) | 3.78 (.65) | ***2.69 (.38)*** |
| Female | 3.63 (.89) | 3.93 (.68) | 3.75 (.82) | ***2.42 (.60)*** |
| Age | -.01 | -.14 | -.012 | -.01 |
| Country of Origin |  |  |  |  |
| Russian | 3.93 (.59) | 4.21 (.47) | 4.22 (.53) | 2.26 (.47) |
| Ukrainian & Belarusian | 3.90 (.58) | 4.34 (.42) | 4.24 (.51) | 2.23 (.53) |
| Time social distancing |  |  |  |  |
| No | 3.91 (.55) | 4.26 (.43) | 4.25 (.52) | 2.32 (.48) |
| One month or less | 3.92 (.66) | 4.45 (.30) | 4.43 (.37) | 2.13 (.56) |
| More than one month | 3.94 (.59) | 4.20 (.49) | 4.16 (.56) | 2.18 (.49) |

Welch ANOVA tests were used to adjust unequal variances. ***Bold and italic*** values represent statistically significant at either *p* <.05 or *p* < .01.

*a* = parents’ reported family context variables were used. *b* = adolescents’ reported family context variables were used.
