# Supplementary figures and images for: Psychological distress, employment, and family functioning during the COVID-19 outbreak among recent immigrant families in Israel: Moderating roles of COVID-19 prevalence
Source: PLoS One. 2022 Nov 17;17(11):e0277757. doi: 10.1371/journal.pone.0277757 (PMC9671308; doi:10.1371/journal.pone.0277757)

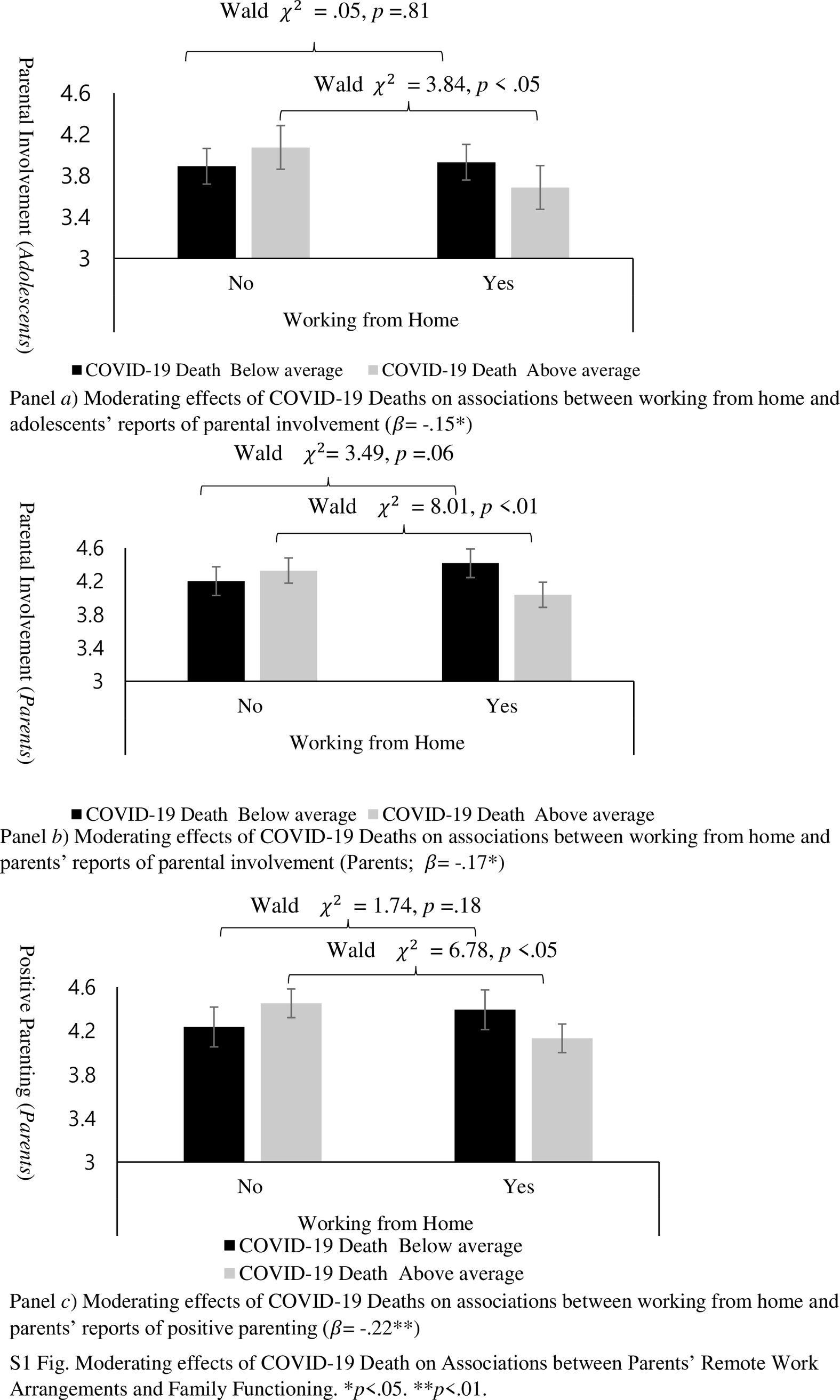

Supplement: S1 Fig — (TIF) [file pone.0277757.s003.tif]
