# Supplementary material for: Psychological distress, employment, and family functioning during the COVID-19 outbreak among recent immigrant families in Israel: Moderating roles of COVID-19 prevalence
Source: PLoS One. 2022 Nov 17;17(11):e0277757. doi: 10.1371/journal.pone.0277757 (PMC9671308; doi:10.1371/journal.pone.0277757)
Supplement: Attachment — Submitted filename: Review PONE-D-22-04347.docx [file pone.0277757.s004.docx]

This contribution is well-written and thorough. However, the main focus – determining how the covid-19 pandemic is related to the functioning of migrant families is not sufficiently well motivated and empirically studied. The main points of criticism are stated below:

- The theoretical arguments that are presented in the paper are generic and applicable to any group/ general population. I miss specific arguments of why should we study the migrant population (beyond the number in the entire population) and how the reaction to the pandemic might differ for this group as compared to native populations.
- Following that, I would suggest including the native population as a comparison group, to disentangle the difference. I am not sure, however, whether the data permits it. Alternatively, one can focus on this one group only and use more migration-specific arguments and indicators to show what is distinct about migrants’ situation.
- The contribution lacks links to the migration literature, and especially – transnationalism. Migrants who have migrated 5 years prior to the study and are in their 40-ies, might still have close ties to the family members living in the country of origin, especially parents (or did the parent generation migrate along?). Stress about elderly parents living abroad, with limited possibility to go and visit can be a stressful event for migrants and can affect their family life during the time of the global pandemic. Does the data set contain any information that would allow disentangling of those mechanisms?
- The authors use convenience sample – how representative is it for the whole migrant population? Is there a way to say anything about that?
- The conclusions about designing possible interventions to deal support migrant families in the time of the pandemic seem also very generic and applicable to general population. Again, what is so unique about this group, that does not apply to native population?
